# Formulation-Dependent Antibacterial Performance: Design and Biomedical Applications

**DOI:** 10.3390/gels12040310

**Published:** 2026-04-03

**Authors:** Ji Won Choi, Younghee Kim, MeeiChyn Goh, Kihak Gwon

**Affiliations:** 1 Department of Biofibers and Biomaterials Science, Kyungpook National University, Daegu 41566, Republic of Korea; cjw5969@gmail.com (J.W.C.); sonho0202@gmail.com (Y.K.); 2 Department of Corporate Support, Healthcare & Spa Industry Promotion Agency, Asan 31442, Republic of Korea; 3 Institute of Medical Imaging, Hengyang Medical School, University of South China, Hengyang 421009, China

**Keywords:** antibacterial materials, formulation architecture, infection microenvironment, antimicrobial resistance, clinical translation

## Abstract

Over the past decade, antibacterial materials have become a promising strategy to address both antibiotic-resistant and biomaterial-associated infections in clinical settings. Despite substantial progress, a gap remains between promising antibacterial performance in vitro and limited therapeutic outcomes in vivo. Herein, we present a mechanistic framework for understanding formulation-dependent antibacterial performance across five representative formulation architectures: nanoparticle-based systems, nanofibrous scaffolds, hydrogel matrices, surface coatings, and vesicular or microencapsulated carriers. We impart how structural organization and delivery dynamics regulate antibacterial mechanisms such as contact-mediated killing, controlled therapeutic release, and reactive oxygen species (ROS) generation and discuss their context-dependent suitability for diverse infection scenarios; these include acute wound infections, biofilm-associated implant infections, and chronic infected wounds. Particular emphasis is placed on factors contributing to the frequent failure of high in vitro log reduction efficacy translating into clinical success, including protein corona formation, biological barrier penetration, and dynamic host–pathogen interactions. Finally, we propose a comparative formulation-selection framework based on infection type, tissue environment, and therapeutic objectives to guide the rational design of next-generation antibacterial materials. This perspective bridges the gap between material innovation and clinical translation by highlighting formulation architecture as a central determinant of antibacterial performance in biomedical applications.

## 1. Introduction

The escalating global health crisis of antibiotic-resistant infections has positioned antibacterial materials as a critical frontier in biomedical research. According to widely cited projections, antimicrobial resistance (AMR) might cause up to 10 million deaths annually by 2050 if left unaddressed, which could result in it potentially surpassing cancer as the leading cause of mortality [1]. In parallel, biomaterial-associated infections, particularly those involving medical implants, catheters, and surgical devices, continue to impose substantial clinical and economic burdens on healthcare systems [2]. Infection rates range from approximately 1–5% for routine procedures and can exceed 20% in high-risk surgeries, leading to prolonged hospitalization, revision surgeries, and increased healthcare costs [3,4]. Consequently, the development of clinically effective antibacterial materials has become essential for advancing modern biomedical technologies, including implant coatings, wound dressings, tissue engineering scaffolds, and drug delivery systems.

Over the past two decades, extensive research efforts have been focused on developing antibacterial materials with incorporated diverse antimicrobial agents such as silver nanoparticles, antibiotics, antimicrobial peptides, and/or metal oxides [5,6,7]. Under controlled laboratory conditions, many of these materials have demonstrated strong antibacterial activity, often achieving multi-log bacterial reduction within relatively short exposure times [8,9]. However, a critical and often underappreciated challenge emerges during clinical translation: antibacterial performance observed in vitro frequently decreases significantly in physiological environments [5,10,11]. This in vitro–in vivo performance gap is one of the most substantial barriers to the clinical translation of antibacterial biomaterials.

The discrepancy between laboratory performance and clinical outcomes is primarily due to multiple formulation- and environment-dependent factors that are often insufficiently addressed in current material design strategies. Upon exposure to biological fluids, antibacterial materials rapidly interact with proteins and biomolecules to form a protein corona that can alter the surface charge, biological recognition, aggregation behavior, and antimicrobial release kinetics [12,13]. In addition, the characteristics of the tissue microenvironment, such as heterogeneous oxygen availability, localized acidic pH in infected wounds, enzymatic degradation, reactive oxygen and nitrogen species, and dynamic immune cell responses, can profoundly influence the functionality of the antibacterial material [14,15]. Furthermore, different infection types present distinct therapeutic challenges: acute wound infections typically require rapid bacterial elimination, whereas chronically infected wounds demand sustained antimicrobial activity within biofilm-rich and poorly vascularized environments [16,17]. Implant-associated infections require both biofilm prevention and long-term antimicrobial protection under host immune surveillance [18]. Despite these complexities, most antibacterial materials continue to be evaluated using simplified in vitro assays, such as planktonic bacterial cultures, which fail to capture biofilm heterogeneity; host–pathogen interactions; and immune-mediated clearance mechanisms [19,20].

A critical though underexplored dimension of antibacterial material design is the profound influence of formulation architecture on antibacterial performance [13,21]. The same antimicrobial agent can exhibit markedly different efficacies depending on whether it is delivered as free molecules, incorporated into nanoparticles, embedded within hydrogels, immobilized on a surface, or encapsulated within vesicles or microcapsules [13,22]. Each formulation platform presents distinct advantages and limitations in terms of loading capacity, release kinetics, spatial distribution, tissue penetration, and interactions with bacterial cells and host tissues [23]. For instance, nanoparticle formulations can achieve high local concentrations and enhanced cellular interactions but may experience rapid clearance or aggregation in biological fluids [13,24]. Although hydrogel systems provide sustained release and moisture retention that is beneficial for wound healing, their ability to penetrate deeply into infection sites is limited [25,26]. Surface coatings enable localized antimicrobial activity suitable for implants but generally lack systemic distribution [27]. Importantly, formulation strategies not only influence delivery efficiency but can also strongly affect predominant antibacterial pathways and exposure modes, including contact killing, oxidative stress generation, membrane disruption, and biofilm penetration [28,29]. In this context, formulation-dependent antibacterial performance refers specifically to how formulation architecture—through its regulation of physicochemical interactions, transport behavior, and mechanistic pathways—acts as a primary determinant of antibacterial efficacy under physiological conditions, rather than a secondary consequence of antimicrobial agent selection alone.

To date, most reviews on antibacterial biomaterials have been primarily focused on specific antimicrobial agents or individual formulation platforms without providing sufficient guidance for rational formulation selection based on clinical infection scenarios [13,30,31,32]. For example, reviews on nanoparticle-based antibacterial systems have extensively characterized physicochemical properties and in vitro bactericidal activity but have rarely addressed how protein corona formation or tissue barrier interactions limit their in vivo efficacy [33]. Similarly, reviews on hydrogel wound dressings have focused on crosslinking strategies and release profiles without systematically linking formulation architecture to infection-specific therapeutic requirements [34,35]. Critically, no existing review has proposed a mechanistic, cross-platform framework that simultaneously addresses formulation-dependent antibacterial mechanisms, in vitro–in vivo performance gaps, and infection-context-specific formulation selection principles. This review therefore addresses these knowledge gaps by providing a mechanistic framework—rather than a descriptive taxonomy—that operationalizes formulation architecture as a central and actionable design variable governing antibacterial performance across diverse infection scenarios. A comparison of representative recent reviews is provided in Table 1.

**Figure 1:**
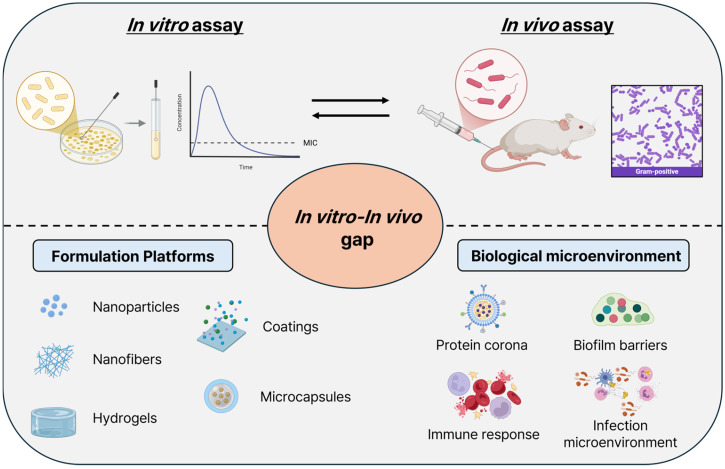
A schematic illustration of the key factors contributing to the in vitro–in vivo performance gap in antibacterial biomaterial development, highlighting the roles of formulation platforms and biological microenvironmental factors. MIC, minimum inhibitory concentration.

To address these objectives, we systematically examine five major formulation platforms, nanoparticles, nanofibers, hydrogels, films and coatings, and microcapsules and vesicles, while evaluating how their inherent structural and physicochemical characteristics modulate antibacterial mechanisms and clinical applicability. For each formulation type, we discuss material design strategies, antibacterial mechanisms, and suitability for distinct infection scenarios, including acute wound infections, chronic infected wounds, and implant-associated infections. Particular emphasis is placed on formulation-related factors contributing to in vitro–in vivo performance gaps, including protein corona formation, tissue penetration barriers, biofilm interactions, and host immune responses. Finally, we propose a comparative framework and formulation-selection guidelines to assist researchers in matching antibacterial material design strategies with specific infection contexts and clinical requirements, thereby advancing the rational development of clinically translatable antibacterial biomaterials. A summary of the key factors contributing to the in vitro–in vivo performance gap for antibacterial materials is provided in Figure 1.

## 2. Design Principles for Formulation-Dependent Antibacterial Performance

The rational design of antibacterial materials extends beyond the intrinsic antimicrobial activity of incorporated agents and must account for how the formulation architecture interacts with complex biological microenvironments [38]. Although many antibacterial systems demonstrate strong bactericidal performance under simplified in vitro conditions, clinical antibacterial efficacy is governed by dynamic interactions among antimicrobial agents, structural organization, biological barriers, and infection-specific pathophysiology [35,39]. Therefore, antibacterial performance should be regarded as a formulation-dependent outcome rather than solely an agent-dependent property. In this context, formulation encompasses material composition, structural configuration, spatial organization, and delivery dynamics [40]. These architectural features regulate antimicrobial exposure mode, transport behavior, retention time, and biological interaction at target infection sites. Effective antibacterial design must therefore integrate the antimicrobial mechanism, infection microenvironment characteristics, and translational constraints within a unified framework. In addition to biological performance, practical translational factors, including sterilization compatibility, manufacturing scalability, and storage stability, must be incorporated as core design considerations because they ultimately determine clinical adoptability regardless of in vitro efficacy.

### 2.1. Architecture–Mechanism Coupling

A fundamental principle in antibacterial formulation design is the coupling between structural organization and the predominant antibacterial mechanism. While antimicrobial agents such as metallic nanoparticles, antibiotics, antimicrobial peptides, and metal oxides exert bactericidal effects through membrane disruption, ROS generation, metabolic inhibition, or intracellular targeting, the relative contribution of these mechanisms in vivo depends strongly on how antimicrobial agents are spatially organized and released within the formulation [41,42]. Surface-immobilized systems predominantly rely on contact-mediated antibacterial activity, whereas matrix-based platforms such as hydrogels or nanofibrous scaffolds enable sustained antimicrobial release and prolonged interfacial exposure. Nanoparticle carriers may enhance membrane interaction and intracellular delivery, thereby shifting effective antibacterial pathways under physiological conditions. Hence, the formulation architecture can directly modulate the antibacterial mechanism and, consequently, the therapeutic outcome.

The chemical properties of antibacterial materials can play a critical role in determining which mechanistic pathways are activated under physiological conditions. The surface charge, for instance, can govern the electrostatic interaction between formulations and negatively charged bacterial membranes: cationic surfaces tend to promote direct membrane disruption, whereas neutral or anionic surfaces may favor alternative mechanisms such as controlled ion release or ROS-mediated oxidative damage [43]. Functional groups such as hydroxyl, carboxyl, and amine moieties can modulate protein adsorption behavior, antimicrobial release kinetics, and bacterial adhesion at material interfaces [44]. Coordinating ligands and redox-active components—such as those found in copper- or zinc-based systems—can facilitate metal ion release under infection-associated acidic conditions, generating localized cytotoxic ion concentrations while also participating in Fenton-type reactions that may amplify ROS production [45]. Furthermore, the redox chemistry of ROS-generating materials, including photocatalytic metal oxides and peroxide-releasing systems, can be strongly influenced by the electronic structure and surface functionalization of the formulation, which in turn determines the rate, duration, and spatial distribution of oxidative stress at infection sites [46]. Understanding and deliberately engineering these chemical properties therefore represents an essential dimension of formulation-dependent antibacterial design. For instance, positively charged silver nanoparticles have been reported to exhibit significantly higher bactericidal activity against both Gram-positive and Gram-negative bacteria compared to their negatively charged counterparts, directly demonstrating how surface charge governs the magnitude of membrane-disruption-driven antibacterial efficacy [47].

Importantly, the predominant antibacterial mechanism is not fixed but can shift depending on the infection microenvironment in which the formulation operates. In acute wound infections characterized by high bacterial load and strong inflammatory activity, nanoparticle-based systems may primarily act through rapid ROS generation and membrane disruption, driven by their high surface area and direct bacterial contact. In contrast, when the same nanoparticle systems encounter biofilm-associated infections, EPS-mediated diffusion restriction and the metabolic dormancy of persister cells can substantially reduce the effectiveness of these rapid-acting mechanisms, necessitating a shift toward sustained ion release or enzyme-triggered antimicrobial activation [48]. Quantitatively, biofilm-associated bacteria can exhibit up to 1000-fold greater resistance to antimicrobial agents compared to their planktonic counterparts, as EPS-mediated diffusion restriction and metabolic dormancy of persister cells collectively reduce the effective intrabiofilm antimicrobial concentration below therapeutic thresholds [49]. Similarly, surface coatings that rely on contact-mediated killing may provide effective prophylaxis against early bacterial adhesion on implant surfaces but may be insufficient for treating established biofilm infections where physical contact between the coating and embedded bacteria is limited [50]. These context-dependent mechanistic shifts underscore why the formulation architecture must be deliberately matched to the dominant biological conditions of the target infection environment, rather than optimized solely for in vitro bactericidal performance, as further discussed in the context of biological barriers and microenvironment matching in Section 2.2.

### 2.2. Barrier Interaction and Microenvironment Matching

Biological transport barriers and infection-specific microenvironments can critically influence antibacterial performance. Upon exposure to physiological fluids, antibacterial materials can undergo protein adsorption, immune recognition, and/or aggregation, which can alter the surface properties, biodistribution, and antimicrobial release behavior [12,51]. In addition, biofilm extracellular polymeric substance (EPS) matrices present particularly formidable barriers, as their complex composition of polysaccharides, proteins, and extracellular DNA can physically restrict antimicrobial diffusion while generating internal gradients in pH, oxygen, and nutrient availability that may promote metabolic heterogeneity and persister cell formation [52]. Quorum sensing-mediated adaptive resistance can further compound these challenges, making biofilm-associated infections substantially more difficult to eradicate than planktonic bacterial infections [19]. Beyond biofilm barriers, heterogeneous tissue structures and dynamic fluid exchange can further restrict antimicrobial penetration and limit the effective drug concentration at infection sites [53].

Infection environments can exhibit substantial heterogeneity in oxygen availability, pH, enzymatic activity, inflammatory status, and biofilm presence [54,55]. For example, chronically infected wounds typically exhibit an alkaline pH ranging from 7.0 to 9.0 and oxygen tension below 1–2%, while elevated protease activity further alters material stability and drug release behavior in vivo [56,57]. Within biofilm structures, internal oxygen gradients can drop to near-anoxic levels within 50–100 μm of the surface, creating metabolically dormant bacterial subpopulations that are substantially less susceptible to conventional antimicrobial treatment [58]. Acute infections typically require rapid antimicrobial exposure within highly vascularized tissues, whereas chronically infected wounds and implant-associated infections may require sustained retention, enhanced barrier penetration, and long-term structural stability. Aligning the formulation properties, such as the size, surface chemistry, release kinetics, and mechanical integrity, with these microenvironmental characteristics is essential for achieving context-appropriate antibacterial performance in vivo.

### 2.3. Spatial Localization and Retention

While barrier interaction addresses the ability of formulations to penetrate biological obstacles and reach infection sites, spatial localization and retention concern the ability to remain confined at those sites once delivered. These two principles are therefore complementary but mechanistically distinct: barrier penetration governs the transport phase of antimicrobial delivery, whereas spatial retention governs the therapeutic residence phase.

Achieving and maintaining therapeutic antimicrobial concentrations at infection sites while minimizing systemic toxicity requires careful control of spatial localization and retention. The formulation architecture determines whether antimicrobial agents remain confined to the material interfaces or undergo rapid clearance through vascular or lymphatic transport [59].

Hydrogels, nanofibrous scaffolds, and surface coatings can act as localized antimicrobial reservoirs, thereby enabling prolonged exposure at infection interfaces [25]. In contrast, freely diffusing nanoparticles or soluble antimicrobial agents can be cleared rapidly, which reduces the effective antimicrobial residence time. Incorporating adhesive functional groups, structural matrices, or depot-forming architectures can improve local retention and thus enhance the therapeutic effectiveness, particularly in chronic wound and implant-associated infection scenarios [60].

### 2.4. Dynamic Responsiveness and Host Integration

Infection microenvironments are dynamic, characterized by fluctuations in pH, enzymatic activity, oxidative stress, and immune signaling, which can evolve over time [61]. Incorporating environmentally responsive elements into antibacterial formulations can enable context-dependent antimicrobial activation and thereby improve therapeutic precision while reducing off-target effects.

Equally important is the consideration of host compatibility and biosafety. Excessive antimicrobial release or uncontrolled reactive species generation can damage host tissues and impair healing, whereas materials that modulate inflammatory responses, support immune-mediated bacterial clearance, and/or promote tissue regeneration can enhance the overall therapeutic outcome [62]. In particular, the cytotoxicity profiles, pharmacokinetic behavior, and long-term biosafety of metal-based nanoparticles and ROS-generating systems must be carefully considered as core design parameters, especially for materials intended for implant applications [63]. Balancing antimicrobial potency with favorable host interactions and long-term biosafety is therefore critical for achieving clinically translatable antibacterial performance.

## 3. Nanoparticle-Based Antibacterial Systems

**Figure 2:**
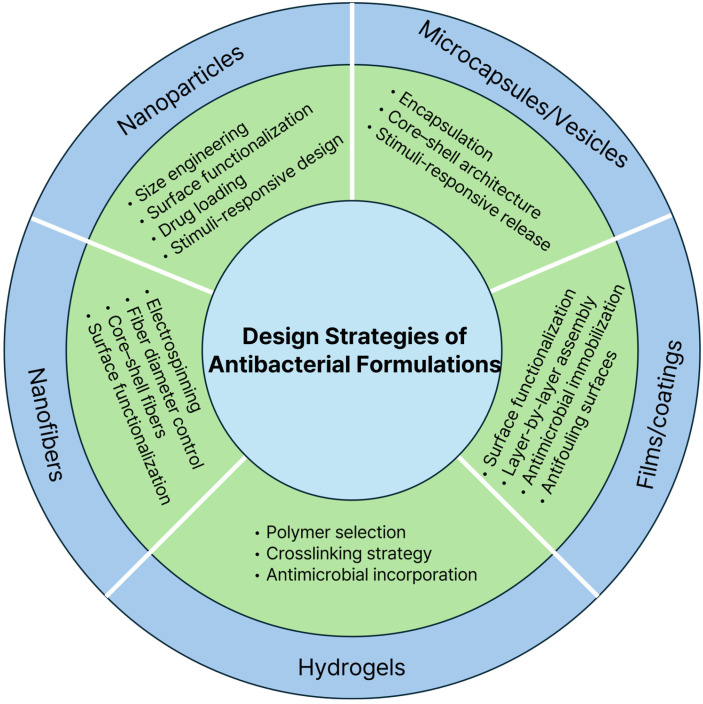
Representative design strategies for five major antibacterial formulation platforms, including nanoparticles, microcapsules/vesicles, films/coatings, hydrogels, and nanofibers.

These are among the most extensively investigated formulation platforms due to their high surface area, tunable physicochemical properties, and ability to achieve localized antimicrobial delivery [11,13]. Nanoparticles engineered from a wide range of materials, including metals and other inorganic materials, polymers, and lipids, can versatilely incorporate antimicrobial agents such as antibiotics, metal ions, antimicrobial peptides, and generating materials [24,29]. Compared to free antimicrobial agents, nanoparticle formulations better protect labile drugs, enhance interactions with bacterial membranes, and increase local antimicrobial concentration at infection sites [64,65]. However, nanoparticle antibacterial performance in physiological environments is strongly influenced by biological interactions such as protein adsorption, aggregation, and immune recognition, thereby highlighting the importance of formulation optimization for maintaining antibacterial efficacy in vivo [12,66]. Based on these design principles, five representative antibacterial formulation platforms—nanoparticles, nanofibers, hydrogels, films/coatings, and microcapsule or vesicle systems—are discussed in the following sections, with Figure 2 presenting a summary of their key design strategies.

### 3.1. Design Strategies

The design of nanoparticle-based antibacterial systems focuses on optimizing size, surface chemistry, drug loading strategies, and release profiles to maximize antibacterial activity while maintaining stability under physiological conditions [64]. Particle size plays a critical role in determining interactions with bacteria, tissue penetration, and clearance behavior [67,68]. Smaller nanoparticles typically exhibit enhanced membrane interaction and tissue penetration, whereas larger particles can provide improved drug loading capacity and prolonged retention at infection sites [67]. However, excessively small nanoparticles can be rapidly cleared by renal filtration or immune-mediated uptake, necessitating careful size optimization depending on the target infection location and administration route [62].

Surface functionalization is another key design strategy [64]. Surface charge and chemical modification influence bacterial adhesion, cellular uptake, and interactions with biological proteins [67,68]. Although positively charged nanoparticles often demonstrate stronger interactions with negatively charged bacterial membranes, they can also exhibit increased adsorption by proteins and potential cytotoxicity [67]. Hydrophilic surface coatings, such as polyethylene glycol or zwitterionic polymers, are frequently employed to reduce adsorption by proteins and improve the colloidal stability of the nanoparticles in biological fluids [69]. In addition, targeting ligands or biofilm-binding moieties can be incorporated to enhance bacterial targeting and improve antimicrobial delivery efficiency [65].

Drug incorporation strategies also significantly influence antibacterial performance. Antimicrobial agents can be physically encapsulated, chemically conjugated, or surface-immobilized on nanoparticles [65]. Encapsulation protects against enzymatic degradation and enables controlled release, whereas surface immobilization can enhance contact-mediated antibacterial activity [67]. Stimulus-responsive nanoparticle systems have also been developed that enable infection-triggered drug release in response to pH changes, enzymatic activity, or ROS present in infected tissues [61,70]. For example, chitosan-coated silver nanoparticles have demonstrated potent antibacterial and antibiofilm activity against uropathogenic *E. coli* at low concentrations, illustrating how surface functionalization with cationic polymers can enhance bacterial membrane targeting and antimicrobial delivery efficiency under physiologically relevant conditions [71].

### 3.2. Antibacterial Mechanisms

Nanoparticle-based antibacterial systems can exert antimicrobial activity through multiple mechanisms that depend on both the nanoparticle material and formulation strategy. One major mechanism involves enhanced interaction with bacterial membranes. Nanoparticles can accumulate at bacterial surfaces, which increases the local antimicrobial concentration and promotes membrane disruption or permeability changes [72,73]. Metallic nanoparticles involving silver or copper can also release antimicrobial ions and generate ROS, resulting in oxidative damage to bacterial membranes, proteins, and DNA [74,75,76].

In addition to direct bactericidal effects, nanoparticle systems can also improve intracellular antimicrobial delivery, particularly against bacteria residing within host cells [65,77]. Nanoparticles can facilitate antibiotic transport across cellular membranes, which increases intracellular drug accumulation and improves bacterial eradication efficiency [78,79]. Furthermore, nanoparticle formulations can improve biofilm penetration by facilitating transport through EPS matrices, although biofilm heterogeneity and density may still limit complete bacterial eradication [80,81].

Importantly, nanoparticle antibacterial mechanisms are strongly influenced by biological environments [59]. Protein adsorption and immune interactions can alter nanoparticle surface properties, thereby affecting bacterial targeting and antimicrobial release behavior [62,66]. Moreover, aggregation in biological fluids can reduce the effective surface area and antimicrobial availability. Therefore, the nanoparticle antibacterial mechanism must be considered in the context of formulation stability and biological interactions.

### 3.3. Biomedical Applications

Nanoparticle-based antibacterial systems have been widely explored across multiple biomedical infection applications, including wound infection treatment, implant-associated infection prevention, and systemic infection therapy [13,82,83]. In acute wound infections, nanoparticles can provide rapid antimicrobial action through high local drug concentrations and enhanced bacterial membrane interaction [82,84]. In chronically infected wounds, nanoparticle systems can be incorporated into secondary formulations such as hydrogels or dressings to enable sustained antimicrobial delivery while maintaining the wound moisture balance [16,33].

For implant-associated infections, nanoparticle-based coatings and surface modifications have been developed that prevent bacterial adhesion and biofilm formation [83,85]. In addition, nanoparticles have been investigated for targeted antimicrobial delivery to infection sites through systemic administration, although biological clearance and off-target accumulation remain major challenges for clinical translation [86,87]. For example, silver nanoparticles incorporated into hydrogel dressings have demonstrated effective reduction in *S. aureus*-infected wound bacterial burden and accelerated wound closure in murine infection models, supporting their translational potential for clinical wound management in cases where high local antimicrobial concentration is required [84].

Despite their promising antibacterial performance in controlled experimental settings, clinical translation of nanoparticle antibacterial systems remains limited [59,88]. Challenges that include protein corona formation, immune clearance, long-term toxicity concerns, and large-scale manufacturing complexity must be addressed to improve clinical translation success [12,89,90]. Continued development of stable, biocompatible, and environment-responsive nanoparticle formulations should enhance their clinical potential for antibacterial therapy.

## 4. Nanofiber-Based Antibacterial Systems

The unique structural properties of nanofibers, including their high surface-to-volume ratio and architectural resemblance to the native extracellular matrix (ECM), position them as powerful platforms for localized and sustained antibacterial applications [91,92]. Nanofibers are typically fabricated using electrospinning or related fiber-forming techniques, which facilitates the incorporation of a wide range of antimicrobial agents, including antibiotics, metallic nanoparticles, antimicrobial peptides, and natural antibacterial compounds [93,94]. Compared with nanoparticle-based systems, nanofibers provide structural advantages such as high porosity, interconnected fiber networks, and controllable fiber diameter, which collectively facilitate cell infiltration, fluid exchange, and sustained antimicrobial exposure at infection sites [95]. These properties make nanofiber systems particularly attractive for applications in wound dressings, tissue engineering scaffolds, and surface antibacterial barriers [96,97].

### 4.1. Design Strategies

The design of nanofiber-based antibacterial systems has been primarily focused on polymer selection, fiber diameter control, drug incorporation strategies, and fiber surface functionalization [91]. Both natural polymers, such as chitosan, collagen, and gelatin, as well as synthetic polymers, including polycaprolactone, poly(lactic acid), and poly(lactic-co-glycolic acid) (PLGA), are widely used to fabricate antibacterial nanofibers [98]. Natural polymers often provide intrinsic biocompatibility and bioactivity, whereas synthetic polymers offer tunable mechanical properties and degradation rate. Blending natural and synthetic polymers is frequently employed to balance biological compatibility and mechanical stability [99].

Fiber diameter is another critical design parameter [100]: a smaller one increases the surface area and drug release rate, while a larger one improves the mechanical strength and prolongs the release duration [101]. Fiber alignment and network density also influence antimicrobial delivery and tissue integration [95]. Random fiber networks typically provide isotropic barrier properties, whereas aligned fibers can guide cell migration and tissue regeneration.

Antimicrobial agent incorporation can be achieved through blending, coaxial electrospinning, or surface immobilization [102]. Although blended nanofibers are easy to fabricate and provide relatively uniform drug distribution, they can also exhibit initial burst release. Core–shell nanofiber structures can provide sustained drug release and improve the protection of labile antimicrobial agents [103]. Moreover, surface functionalization can provide direct contact with bacteria, thereby enhancing contact-mediated antibacterial activity [91]. In addition, multifunctional nanofiber systems incorporating multiple antimicrobial agents or combining antimicrobial and wound healing factors have been increasingly explored [99]. For example, core/shell alginate/poly(ε-caprolactone) nanofibers incorporating gentamicin as an antibacterial agent have demonstrated substantially reduced initial burst release compared to conventional hydrogel film formulations, illustrating how core–shell fiber architectures can enable more controlled and sustained antimicrobial delivery [104].

### 4.2. Antibacterial Mechanisms

Nanofiber-based antibacterial systems primarily function through sustained antimicrobial release and contact-mediated antibacterial activity [102]. Due to their high surface area and interconnected porous structure, nanofibers can maintain the localized antimicrobial concentration at the infection site over an extended period. This sustained-release behavior is particularly beneficial for preventing bacterial regrowth and maintaining the antibacterial response in the infected tissues.

In addition to slow-release-based antibacterial activity, nanofiber surfaces can directly interact with bacterial cells [105]. Antimicrobial agents immobilized on the fiber surfaces can induce membrane disruption or metabolic inhibition upon contact with bacteria. In some cases, altering the nanofiber structural properties (e.g., surface roughness and hydrophilicity) can influence the local microenvironment, thereby mitigating bacterial adhesion and colonization [106].

Nanofiber systems can also improve antibacterial performance in biofilm-associated infections. Although the porous fiber network can facilitate diffusion of antimicrobial agents into biofilm structures, complete eradication of the latter remains challenging due to biofilm heterogeneity and protective ECMs [107,108]. Importantly, nanofiber antibacterial performance can be influenced by biological fluid interactions and material degradation behavior, which affects the antimicrobial release profile and localized concentration [12].

### 4.3. Biomedical Applications

Nanofiber-based antibacterial systems have been extensively investigated for wound healing and infection prevention applications [96,109,110]. In acute wound infections, nanofiber dressings can provide immediate antibacterial protection while maintaining a moist wound environment conducive to tissue repair, while in chronically infected wounds, they can provide sustained antimicrobial release while supporting cell infiltration and tissue regeneration [16].

Nanofiber systems have also been explored as antibacterial coatings or barrier layers for medical devices and implant surfaces [111,112]. Their high surface area enables effective antimicrobial loading and localized antibacterial activity, albeit their long-term mechanical stability and adhesion to device surfaces remain important considerations.

Despite their promising antibacterial performance in experimental settings, clinical translation of nanofiber antibacterial systems requires further optimization [113,114]. Challenges that include large-scale manufacturing consistency, sterilization stability, long-term storage stability, and mechanical durability under physiological conditions must be addressed. Continued development of multifunctional and environment-responsive nanofiber systems should further improve their clinical potential as an antibacterial therapy for infection prevention and mitigation. For example, chitosan-based nanofiber scaffolds incorporating silver nanoparticles have demonstrated synergistic antibacterial activity against *S. aureus* and *E. coli* alongside accelerated wound closure in preclinical infection models, supporting the translational potential of nanofiber-based antibacterial systems for infected wound management as multifunctional wound dressings [115].

## 5. Hydrogel-Based Antibacterial Systems

Beyond fiber-based architectures, three-dimensional hydrogel networks offer a distinct set of functional advantages, including tunable mechanical properties, high water content, and inherent biocompatibility, which make them particularly suited for simultaneous antimicrobial delivery, microenvironment modulation, and tissue integration. [25,26]. Hydrogels comprise three-dimensional hydrophilic polymer networks capable of retaining a large amount of water, which enables them to mimic the physical and biochemical characteristics of the native ECM [116,117]. Due to their high water content, tunable mechanical properties, and ability to encapsulate a wide range of antimicrobial agents, hydrogels have been widely explored for wound healing, implant coatings, and localized infection control [7,26]. Compared to nanoparticle and nanofiber systems, hydrogels offer distinct advantages by maintaining prolonged contact with infected tissues and providing sustained antimicrobial exposure, while simultaneously supporting tissue regeneration processes [52].

### 5.1. Design Strategies

The design of hydrogel-based antibacterial systems is primarily focused on polymer selection, crosslinking strategy, mechanical property optimization, and antimicrobial incorporation methods [26]. Hydrogel networks can be constructed from diverse polymeric building blocks, ranging from naturally derived biomacromolecules to fully synthetic polymers with tunable physicochemical properties [37,118]. Naturally derived polymers often provide intrinsic bioactivity and cell-interactive features, whereas synthetic systems enable precise control over the mechanical strength, degradation kinetics, and network architecture.

Crosslinking strategies play a critical role in determining the hydrogel structure and antibacterial performance [26]. Physical crosslinking methods, such as ionic interactions and hydrogen bonding, afford injectable or self-healing hydrogels with dynamic network properties [119,120]. Chemical crosslinking methods, including covalent bond formation and photo-crosslinking, provide improved mechanical stability and long-term structural integrity. Hybrid crosslinking approaches are being increasingly used to combine mechanical robustness with environmental responsiveness [121].

Antimicrobial incorporation strategies include direct drug encapsulation, nanoparticle loading, and polymer conjugation [33]. Encapsulation of antibiotics or antimicrobial peptides enables their sustained release through diffusion and matrix degradation [122]. Incorporating antimicrobial nanoparticles can enhance the antibacterial potency while maintaining the localized antimicrobial concentration [33]. In addition, polymer functionalization, such as adding catechol or phenolic groups, can provide adhesive properties and improve retention at the infection site [47]. For example, a copper-based metal–organic framework (MOF)-embedded dual-crosslinked alginate hydrogel has demonstrated enhanced antibacterial activity through the combined effects of sustained metal ion release and structural stability, illustrating how hybrid crosslinking strategies and functional material incorporation can synergistically improve antibacterial performance under physiologically relevant conditions [123].

### 5.2. Antibacterial Mechanisms

Hydrogel-based antibacterial systems primarily exert antimicrobial effects through sustained release of antimicrobial agents and localized microenvironment modulation [38]. The hydrogel matrix can act as a reservoir that maintains the antimicrobial at a therapeutic concentration at the infection site over an extended period, which is particularly beneficial for treating chronic infections and preventing bacterial recolonization.

In addition to sustained antimicrobial release, the network composition and porosity of the hydrogel can be altered to modulate the local infection microenvironment by maintaining moisture at the wound site, regulating the local pH, and supporting oxygen diffusion [25,26]. Moreover, ROS-generating components or metal ions can also be incorporated into the hydrogel to enhance direct antibacterial activity [124].

The hydrogel’s structural properties also influence bacterial adhesion and biofilm formation. Depending on the mesh size, the hydrated polymer network can limit bacterial penetration while simultaneously enabling diffusion of antimicrobial agents [52,125]. However, excessive network density could restrict drug diffusion and reduce antibacterial effectiveness, highlighting the importance of balancing structural stability and antimicrobial transport.

Hydrogel antibacterial performance is also influenced by biological interactions, including enzymatic degradation, protein adsorption, and immune cell infiltration [12]. These factors can affect the hydrogel’s stability, antimicrobial release kinetics, and overall antibacterial efficacy under physiological conditions.

### 5.3. Biomedical Applications

Hydrogel-based antibacterial systems have already been extensively applied in wound healing, localized infection treatment, and implant-associated infection prevention [25,126,127]. In acute wound infections, hydrogels can provide immediate antimicrobial delivery while maintaining hydration and supporting early tissue repair, whereas in chronically infected wounds, they provide sustained antimicrobial release and can help disrupt biofilm-associated infections while maintaining a favorable tissue regeneration environment.

Hydrogels have also been explored as coatings or interfacial layers for medical implants to reduce bacterial adhesion and biofilm formation [7,128]. Injectable antibacterial hydrogels have been developed that provide minimally invasive delivery and localized antimicrobial retention for the localized treatment of infections in irregular tissue wounds [120].

Despite the promising therapeutic potential of hydrogel-based antibacterial systems, several challenges remain for their clinical translation; these include mechanical durability under physiological stress, long-term storage stability, sterilization compatibility, and large-scale manufacturing reproducibility, which must be addressed [25,129]. Future hydrogel design strategies should focus on multifunctional systems capable of combining antimicrobial activity with immune modulation, biofilm disruption, and tissue regeneration. For example, an injectable collagen-based hydrogel incorporating in situ generated silver nanoparticles has demonstrated broad-spectrum antibacterial activity and significantly accelerated wound healing in diabetic full-thickness wound models, supporting its translational potential for localized infection treatment and tissue regeneration [130].

## 6. Film and Coating-Based Antibacterial Systems

These systems provide surface-localized antimicrobial protection, which is particularly important for preventing device-associated infections and biofilm formation on medical materials [7,131]. Unlike nanoparticle, nanofiber, or hydrogel systems that primarily function through bulk antimicrobial delivery, film and coating-based antibacterial formulations are designed to regulate interfacial interactions between the biomaterial’s surface, bacterial cells, and host tissues [27]. Antibacterial films and coatings can be fabricated as polymer thin films, layer-by-layer assemblies, covalently grafted antimicrobial coatings, or composite coatings incorporating antimicrobial nanoparticles or drugs [132]. These systems have been widely investigated for applications involving implantable devices, catheters, surgical instruments, and wound-contact materials, where the prevention of initial bacterial adhesion is critical [7,131].

### 6.1. Design Strategies

The design of antibacterial films and coatings is primarily focused on surface chemistry modification, coating stability, antimicrobial immobilization strategies, and controlled antimicrobial release. Surface chemical modification can regulate bacterial adhesion and protein adsorption by controlling the surface charge, hydrophilicity, and surface energy [12,31]. Hydrophilic and zwitterionic coatings are commonly used to reduce nonspecific protein adsorption and bacterial attachment, whereas antimicrobial functional coatings actively kill bacteria upon contact [133].

Coating stability is another critical design parameter, particularly for long-term implant applications [31]. Covalent surface grafting methods can provide strong adhesion between the coating layers and substrate materials, thereby reducing coating delamination during implantation and long-term use [134]. Layer-by-layer assembly techniques provide precise control over coating thickness and antimicrobial loading, while also allowing for the incorporation of multifunctional layers with antifouling and antibacterial functionality [135].

Antimicrobial incorporation strategies for coatings include surface immobilization, controlled surface release, and composite coating design. Surface immobilization enables direct contact-mediated antibacterial activity without significant drug diffusion into the surrounding tissues [136]. Controlled release coatings can provide sustained antimicrobial release at the implant interfaces [15], while composite coatings containing antimicrobial nanoparticles or metal ions can enhance antibacterial potency while maintaining localized antimicrobial activity [137]. In addition, stimuli-responsive coatings that can respond to pH changes, enzymatic activity, or bacterial metabolic byproducts have been explored to enable infection-triggered antimicrobial release [138]. For example, a zwitterionic polymer-based antifouling coating has demonstrated significant reduction in bacterial adhesion and protein adsorption on implant surfaces, illustrating how surface chemical modification can effectively regulate interfacial interactions under physiologically relevant conditions [139].

### 6.2. Antibacterial Mechanisms

Film and coating-based antibacterial systems primarily function by preventing bacterial adhesion, contact-mediated bacterial killing, and localized antimicrobial release. Antifouling coatings can reduce initial protein adsorption and bacterial attachment, thereby preventing early-stage biofilm formation [107,140], while contact-active antibacterial coatings can disrupt bacterial membranes or interfere with bacterial metabolic processes upon direct contact with bacterial cells [50,141].

Controlled antimicrobial release coatings can maintain localized antimicrobial concentrations at the material surfaces that prevent bacterial colonization and biofilm maturation [7]. In some systems, metal ion release or ROS generation at the coating interfaces can further enhance antibacterial activity [142,143]. Importantly, coating-based antibacterial performance is strongly influenced by long-term coating stability in terms of mechanical wear and biological surface interactions, including protein adsorption and immune responses [12].

Biofilm prevention is a particularly important mechanism for coating-based antibacterial systems [19,83]. Once mature biofilms have become established on implant surfaces, ensuring total bacterial eradication becomes extremely difficult. Therefore, coating strategies are often focused on preventing initial bacterial adhesion and early biofilm formation rather than eradicating mature biofilms.

### 6.3. Biomedical Applications

Film and coating-based antibacterial systems are widely applied in medical devices and implantable materials, including orthopedic implants, cardiovascular devices, catheters, and surgical instruments. When used with implants, antibacterial coatings can provide long-term protection against bacterial colonization and biofilm formation [27]. Catheter coatings have been widely investigated to reduce catheter-associated urinary tract and bloodstream infections [144].

Antibacterial films are also used in wound-contact materials and protective barrier layers, where localized antimicrobial protection is required rather than systemic antimicrobial exposure. In addition, antibacterial coatings have been explored for dental implants and tissue-contact materials, where long-term bacterial control is critical [7]. For example, antibacterial coatings incorporating silver or antibiotic-releasing layers have demonstrated effective prevention of bacterial colonization and biofilm formation on implant and catheter surfaces in preclinical and clinical settings, supporting their translational potential for device-associated infection prevention [145].

Despite extensive research, the clinical translation of antibacterial coatings remains challenging due to the requirements for long-term stability, coating durability under physiological mechanical stress, sterilization compatibility, and regulatory considerations for implantable materials [129,146]. To improve long-term infection prevention, future coating design strategies should be focused on multifunctional coatings that combine antifouling, antibacterial, and immune-modulating properties.

## 7. Microcapsule- and Vesicle-Based Antibacterial Systems

Encapsulation within microcapsules and vesicular carriers introduces a fundamentally different design logic: one that prioritizes structural protection of antimicrobial agents, programmable release kinetics, and stimuli-triggered payload deployment in the context of infected biological environments [147,148]. Unlike nanoparticle or hydrogel systems that often rely on passive diffusion or structural retention, microcapsule- and vesicle systems enable spatial and temporal control of antimicrobial release [21]. These systems can be fabricated as polymeric microcapsules, liposomes, polymeric vesicles, or hybrid core–shell structures that can encapsulate antibiotics, antimicrobial peptides, metal ions, and/or enzyme-responsive antimicrobial agents [149]. The ability to protect antimicrobial agents from premature degradation while enabling targeted or environment-responsive release makes these systems particularly attractive for treating biofilm-associated and chronic infections [147,150].

### 7.1. Design Strategies

The design of microcapsule- and vesicle-based antibacterial systems is focused on shell material selection, encapsulation efficiency, release triggering mechanisms, and structural stability. Organic polymeric materials such as alginate and chitosan, along with synthetic copolymers such as PLGA, are commonly used to fabricate microcapsules, as well as lipid bilayers and polymeric membranes typically used for vesicle systems [151]. Shell permeability and mechanical stability must be carefully controlled to balance antimicrobial protection and controlled release [152].

Encapsulation strategies include single-agent loading, co-encapsulation of multiple antimicrobial agents, and incorporating enzyme-responsive or pH-responsive release mechanisms [153]. Stimuli-responsive systems are particularly attractive for infection-targeting drug delivery [154,155]. Infection sites often exhibit acidic pH, elevated enzyme levels, and increased ROS, which can be exploited to trigger antimicrobial release specifically at the infected site.

Surface functionalization can also be applied to microcapsules or vesicles to improve bacterial targeting or biofilm penetration [156]. In addition, hybrid delivery systems combining microcapsules or vesicles with secondary matrices, such as hydrogels or coatings, have been explored to improve local retention and therapeutic duration [157]. For example, bioactive hydrogel microcapsules capable of controlled release and reloading of growth factors have demonstrated dynamic regulation of the local microenvironment and sustained bioactivity, illustrating how microcapsule-based systems can achieve programmable and responsive payload delivery under physiologically relevant conditions [158].

### 7.2. Antibacterial Mechanisms

Microcapsule- and vesicle-based antibacterial systems primarily function by controlling antimicrobial release and protecting antimicrobial agents from premature degradation [152]. Encapsulation can improve the stability of antibiotics or antimicrobial peptides under physiological conditions, thereby enabling sustained antimicrobial activity at infection sites.

Stimuli-responsive release mechanisms enable antimicrobial delivery specifically under infection-related conditions [70]. For example, pH-sensitive or enzyme-responsive shell degradation can trigger antimicrobial release at the infected site while minimizing off-target drug exposure. This targeted release behavior can improve antimicrobial efficiency while reducing systemic toxicity.

In addition, microcapsule and vesicle systems can improve antimicrobial penetration into biofilms [159,160], while controlled release within the biofilm microenvironment can help maintain effective antimicrobial concentrations over extended periods. However, biofilm heterogeneity and diffusion barriers still present significant challenges for total bacterial eradication.

Biological interactions, including protein adsorption, immune recognition, and enzymatic degradation, can influence the capsule or vesicle’s stability and drug release kinetics [12]. Therefore, maintaining structural stability while enabling controlled antimicrobial release remains a key design consideration.

### 7.3. Biomedical Applications

Microcapsule- and vesicle-based antibacterial systems have been explored for treating chronic, biofilm-associated, and localized infections [16]. In chronically infected wounds, microcapsule-based delivery systems can provide sustained antimicrobial release while protecting the latter from degradation in harsh wound environments. These systems have also been investigated for targeted antimicrobial delivery to implant-associated infections and localized tissue infections. Injectable microcapsule systems ensure minimally invasive antimicrobial delivery to irregular tissue injuries [161], while vesicle-based systems, such as liposomes, can be used for systemic antimicrobial delivery with improved pharmacokinetics and reduced systemic toxicity [162,163]. For example, phage–liposome nanoconjugates have demonstrated enhanced biofilm eradication in orthopedic infection models, supporting their translational potential for treating biofilm-associated and implant-related infections through advanced vesicle-based delivery strategies [164].

Despite their promising therapeutic potential, several translational challenges remain [165]. Structural stability during storage and sterilization, large-scale manufacturing reproducibility, and regulatory complexity associated with advanced drug delivery systems must be addressed. Future research should be focused on multifunctional microcapsule and vesicle systems capable of combining infection-responsive antimicrobial release, immune modulation, and biofilm-targeted therapy. Table 2 summarizes the key advantages, primary limitations, dominant antibacterial mechanisms, and representative clinical applications of the five major antibacterial formulation platforms discussed in this review.

## 8. The Integrated Formulation-Selection Framework

**Figure 3:**
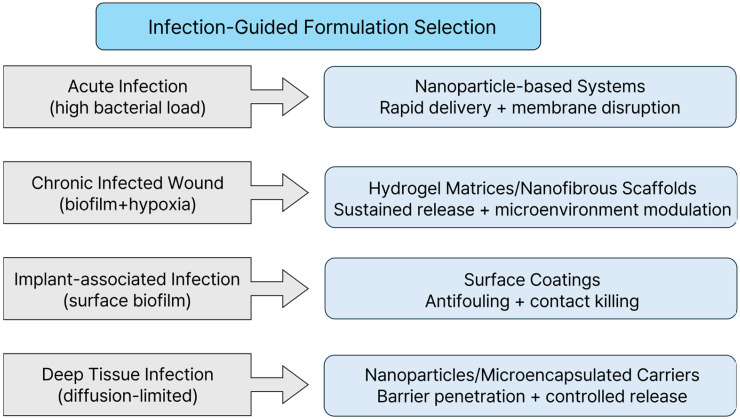
An infection-guided formulation-selection framework mapping four clinically relevant infection scenarios to their preferred formulation architectures and dominant antibacterial mechanisms.

Having examined the antibacterial performance of five representative formulation architectures across Section 3, Section 4, Section 5, Section 6 and Section 7, we here integrate the design principles established in Section 2—architecture–mechanism coupling, barrier interaction and microenvironment matching, spatial localization and retention, and dynamic responsiveness and host integration—into a comparative formulation-selection framework. A high in vitro log reduction value does not necessarily guarantee clinical success. Instead, effective antibacterial performance depends on appropriate matching between the formulation design and the infection context [35,59]. As shown in Figure 3, the therapeutic requirements for antibacterial formulations vary substantially depending on the infection environment.

### 8.1. Microenvironment Matching as the Baseline Selection Criterion

The infection microenvironment matching principle establishes that formulation selection must begin with thoroughly characterizing the target infection microenvironment. Infection scenarios differ substantially in terms of bacterial load, vascularization, pH, oxygen availability, biofilm presence, and immune activity; these differences impose distinct therapeutic requirements that no single formulation architecture can universally satisfy.

Acute infections, characterized by a high bacterial load and strong inflammatory responses in well-vascularized tissues, require formulations capable of rapid antimicrobial delivery and high local concentration. A chronically infected wound, presenting as a persistently inflamed, hypoxic, and biofilm-rich microenvironment, demands sustained antimicrobial exposure, biofilm penetration capability, and microenvironment modulation. Implant-associated infections require surface stability, biofilm resistance, and long-term antimicrobial retention [16,83]. Treating infection microenvironment matching as the primary selection criterion thus provides the baseline from which subsequent formulation decisions can be rationally made.

### 8.2. Barrier Penetration Capability as a Critical Selection Factor

The barrier interaction principle directly influences the formulation selection for infections involving diffusion-limited microenvironments. Biofilm-associated infections represent the most clinically significant barrier challenge because EPS matrices substantially restrict antimicrobial penetration and reduce local therapeutic concentrations [19,34]. Meanwhile, protein corona formation, tissue matrix barriers, and immune clearance mechanisms further limit effective antimicrobial delivery in vivo [12].

Nanoparticle-based systems offer advantages in the context of barrier penetration due to their tunable size, surface chemistry, and capacity for functionalization to reduce nonspecific protein adsorption. Moreover, stimuli-responsive nanoparticles can further enhance penetration by adaptation of their physicochemical properties in response to infection-associated signals [80]. Conversely, matrix-based systems such as hydrogels and nanofibrous scaffolds may provide limited active penetration but compensate for this through sustained, localized release at the infection site. Formulation selection for biofilm-associated and diffusion-limited infections should therefore prioritize architectures that maximize barrier penetration without compromising retention at the infection site.

### 8.3. Spatial Localization and Retention in Formulation Decision-Making

The spatial localization and retention principle governs formulation selection in infection scenarios requiring prolonged antimicrobial exposure at a defined anatomical site. While barrier penetration is critical for biofilm disruption, excessive diffusion of antimicrobial agents away from the infection site can reduce the therapeutic concentration and increase the risk of systemic toxicity [87].

Hydrogel matrices, nanofibrous scaffolds, and surface coatings function as localized antimicrobial reservoirs that maintain sustained exposure at the infection interface. Injectable hydrogels and microcapsule-based carriers are particularly suited for deep tissue infections where localized retention and controlled release are the predominant therapeutic objectives [120]. In contrast, although freely diffusing nanoparticles may be advantageous in acute infections requiring rapid distribution, they are less appropriate for scenarios demanding prolonged localized exposure. Balancing penetration and retention through formulation architecture engineering is therefore a central decision criterion in this selection framework.

### 8.4. Architecture–Mechanism Coupling and Dynamic Responsiveness

The architecture–mechanism coupling principle establishes that the predominant antibacterial mechanism is not solely determined by antimicrobial agent selection but also strongly regulated by the formulation architecture. Surface coatings primarily provide contact-mediated killing or antifouling mechanisms, while nanoparticle carriers enable intracellular delivery and localized concentration enhancement. Hydrogel matrices and encapsulated systems facilitate sustained or stimuli-responsive release, whereas nanofibrous scaffolds combine structural retention with interfacial antibacterial interactions [155].

For implant infection prevention, contact-active coatings offer continuous surface protection without relying on drug release kinetics, while for chronic wound treatment, sustained-release or infection-responsive systems better address the temporal dynamics of wound healing and bacterial persistence. The dynamic responsiveness principle further supports the integration of stimuli-responsive elements such as pH-sensitivity or enzyme-triggered release, particularly in infection environments characterized by temporal fluctuations in the microenvironment conditions.

### 8.5. Host Compatibility and Translational Feasibility

Regardless of in vitro antibacterial efficacy, formulations that induce excessive cytotoxicity, impair tissue regeneration, and/or elicit adverse immune responses are unlikely to achieve clinical translation [165]. Similarly, materials with poor manufacturing scalability, sterilization incompatibility, and/or regulatory complexity provide substantial barriers to clinical implementation [129].

Surface coatings and nanofibrous scaffolds generally offer favorable biocompatibility profiles due to their structural confinement of antimicrobial components. Hydrogel matrices can simultaneously support tissue regeneration while providing antimicrobial functionality, which makes them particularly suitable for wound healing applications. Nanoparticle-based systems require careful surface engineering to minimize cytotoxicity and immune activation. Clinical translational feasibility considerations, including manufacturing complexity and storage stability, should therefore be weighed against biological performance when selecting formulations for specific clinical applications.

### 8.6. Integrated Decision Framework

Based on the four design principles established in Section 2, an integrated decision framework for antibacterial formulation selection can be proposed. This framework does not assume universal superiority of any single material platform but instead guides selection based on which architecture best satisfies the predominant design constraints present in a given infection scenario.

Thus, the following general decision rules can be constructed by considering this principles-based framework: (1) infection microenvironment matching determines the baseline formulation category suitable for a given infection type; (2) barrier penetration capability is critical for biofilm-associated and diffusion-limited infections; (3) spatial localization and retention are predominant considerations for chronic or open-tissue environments requiring prolonged exposure; (4) architecture–mechanism coupling guides selection based on the desired predominant antibacterial mechanism; (5) host compatibility and translational feasibility ultimately determine clinical viability.

Collectively, these principles reinforce that antibacterial efficacy should be understood as a function of formulation–microenvironment interactions rather than intrinsic antimicrobial potency alone. By emphasizing formulation–microenvironment matching over isolated material optimization, this framework is essential for bridging the persistent gap between promising laboratory performance and clinically effective antibacterial therapy [59]. The key evaluation metrics, preferred formulation architectures, dominant antibacterial mechanisms, and relevant design principles for each infection scenario are summarized in Table 3.

## 9. Challenges and Future Perspectives

Although the formulation-selection framework presented in Section 8 provides a structured basis for rational antibacterial material design, multiple scientific, translational, and regulatory challenges continue to limit the clinical adoption of antibacterial biomaterials. While many of them have demonstrated strong antimicrobial activity under controlled experimental conditions, maintaining consistent antibacterial performance in complex physiological environments remains a fundamental limitation [35]. Biological interactions such as protein adsorption, immune cell recognition, enzymatic degradation, and dynamic fluid exchange can significantly alter the material structure, antimicrobial release behavior, and antibacterial mechanism [12]. Therefore, future antibacterial material design should be focused on improving stability and functionality under physiologically relevant conditions.

One major challenge is the need to better understand and control interactions between antibacterial materials and biological systems. Upon exposure to biological environments, antibacterial materials can undergo rapid surface and structural modifications that influence their delivery and bacterial targeting. In particular, protein adsorption and immune system interactions can affect the material biodistribution, clearance rate, and therapeutic retention of the antimicrobial at the infection site [12]. Developing materials that can maintain their structural and functional integrity while responding appropriately to biological microenvironments is critical for improving clinical translation.

A critical but underappreciated challenge in antibacterial biomaterial development is the translational gap arising from the widespread reliance on simplified in vitro evaluation models. Standard antibacterial assessments based on planktonic MIC measurements and log reduction assays fail to capture key physiological variables present at infection sites, including biofilm heterogeneity, protein corona formation, immune cell interactions, and dynamic fluid exchange [23]. Three-dimensional biofilm models and animal infection models more closely approximate in vivo conditions; however, they remain underutilized in preclinical evaluation pipelines. Common animal models for evaluating antibacterial biomaterials include murine full-thickness wound models, subcutaneous implant infection models, and rat osteomyelitis models, but differences in skin thickness, vascularization, immune response kinetics, and wound microenvironment between rodents and humans can limit their predictive accuracy for clinical outcomes [166,167]. A representative example of this translational challenge is silver nanoparticle-based antibacterial systems, which consistently demonstrate potent multi-log bactericidal activity against planktonic bacteria in vitro but frequently exhibit substantially reduced efficacy in vivo due to protein corona formation, rapid systemic clearance, and dose-dependent cytotoxicity [168]. These observations collectively underscore the need for more biologically representative preclinical evaluation frameworks that incorporate three-dimensional biofilm models, relevant animal infection models, and physiologically accurate testing conditions as standard components of antibacterial biomaterial development.

The treatment of biofilm-associated infections remains one of the most persistent and clinically significant challenges in antibacterial biomaterial development. Biofilms provide strong protection against antimicrobial agents through their EPS matrices, metabolic heterogeneity, and adaptive resistance mechanisms [19,53]. The EPS matrix can physically restrict antimicrobial diffusion, while internal gradients in pH, oxygen, and nutrient availability may promote persister cell formation and metabolic dormancy, rendering bacterial subpopulations substantially less susceptible to conventional antimicrobial treatment. Quorum sensing-mediated adaptive resistance can further compound these challenges, and even formulations demonstrating effective planktonic bacterial killing in vitro may frequently fail to achieve therapeutic concentrations within biofilm structures in vivo. Conventional antibacterial materials often fail to achieve complete biofilm eradication, particularly in chronic infection environments. Future strategies should therefore involve materials capable of simultaneously penetrating the EPS matrix, delivering sustained antimicrobial concentrations to metabolically dormant bacterial subpopulations, and disrupting quorum sensing signaling.

Host toxicity and long-term biosafety represent additional critical challenges that must be addressed for successful clinical translation. Metal-based nanoparticles, including silver-, zinc oxide-, and copper-based systems, can exhibit dose-dependent cytotoxicity and may induce inflammatory responses through ROS-mediated oxidative stress and pro-inflammatory cytokine activation [89]. The pharmacokinetics and biodistribution of nanostructured antibacterial systems require careful evaluation, as systemically circulating nanoparticles may accumulate in off-target organs such as the liver, spleen, and kidneys, raising concerns about hepatotoxicity and nephrotoxicity [169]. For antibacterial materials designed for implant applications, chronic exposure to metal ion release or persistent ROS generation may impair tissue integration and provoke sustained local inflammatory responses over extended implantation periods [170]. Regulatory approval pathways for multifunctional antibacterial biomaterials that combine drug delivery with device functions—such as drug-eluting coatings, nanoparticle-loaded hydrogels, and infection-responsive implant surfaces—remain poorly defined, further complicating clinical translation [171]. Establishing standardized preclinical biosafety evaluation frameworks and engaging with regulatory bodies at early design stages may help accelerate the clinical adoption of next-generation antibacterial biomaterials.

Manufacturing scalability and regulatory translation present additional major barriers to clinical translation [15,165]. Many advanced antibacterial materials involve complex synthesis processes, multi-component formulations, and/or stimuli-responsive mechanisms that may be difficult to reproduce consistently at the industrial scale. In addition, regulatory approval for multifunctional antibacterial biomaterials remains poorly defined, particularly for materials that combine drug delivery with the device function. Simplifying material design while maintaining therapeutic functionality might improve clinical translation feasibility.

Looking forward, the production of next-generation antibacterial materials should be focused on multifunctional and adaptive design strategies. Environment-responsive materials capable of adjusting antimicrobial release based on infection-associated signals such as a pH shift, enzymatic activity, or oxidative stress might improve therapeutic precision [155]. Integrating immune-modulating functions could further enhance host-mediated bacterial clearance while reducing reliance on high-dose antimicrobial agents [165]. Hybrid formulation systems that combine multiple antibacterial mechanisms and delivery platforms—such as nanoparticle-loaded hydrogels or nanofiber–hydrogel composites—could also play an important role by simultaneously addressing penetration, retention, and controlled release requirements, thereby helping overcome biological barriers and improve antibacterial performance under physiological conditions. Another emerging research direction involves designing antibacterial materials that actively modulate the infection microenvironment; rather than focusing solely on bacterial killing, future materials could regulate local immune responses, control inflammatory signaling, and improve tissue regeneration while preventing bacterial growth [56]. This integrated therapeutic approach might be particularly important for treating chronic infected wounds and implant-associated infections.

Finally, the development of clinically translatable antibacterial materials will require closer integration between materials science, microbiology, immunology, and clinical medicine. Collaborative interdisciplinary research will be essential for developing antibacterial materials that are not only effective in the laboratory but also capable of addressing clinically relevant infection challenges. Ultimately, advancing antibacterial materials design from agent-centered optimization toward formulation–microenvironment integrated strategies is essential for closing the gap between laboratory innovation and clinical implementation.

## 10. Conclusions

In this review, we revealed that different formulation platforms offer distinct advantages and limitations depending on the targeted infection scenario. Nanoparticle systems enable rapid antimicrobial delivery and enhanced bacterial interaction, whereas nanofibrous scaffolds provide structural support and sustained local antimicrobial exposure. Hydrogel matrices offer microenvironment regulation and prolonged antimicrobial retention, while surface coatings are particularly effective for preventing surface-associated infections. Vesicular and microencapsulated carriers provide advanced controlled release and infection-responsive antimicrobial delivery capabilities. Therefore, for the rational selection of an antibacterial formulation, one must consider the infection type, biological barriers, and therapeutic goal.

Building on the formulation–microenvironment integrated framework outlined in this review, future antibacterial material development should shift from agent-centered optimization toward formulation–microenvironment integrated design strategies. Integration of multifunctional properties, including infection-responsive antimicrobial release, immune modulation, biofilm disruption, and tissue regeneration, should improve the therapeutic outcomes. In addition, addressing challenges related to manufacturing scalability, regulatory translation, and long-term biological safety is essential for successful clinical implementation.

Overall, advancing antibacterial biomaterials requires a holistic design approach that integrates the antimicrobial mechanism, formulation architecture, biological interactions, and clinical requirements. Applying the formulation-selection framework grounded in design principles and developing clinically translatable multifunctional antibacterial systems detailed herein will be critical for improving infection treatment and prevention in modern biomedical applications.

## Figures and Tables

**Table 1:** Comparison of representative reviews on antibacterial biomaterials published in the last five years, highlighting the unique contributions of the present review in terms of cross-platform mechanistic analysis, in vitro–in vivo performance gap discussion, infection-specific formulation selection, and clinical translation orientation.

Main Focus	Platforms Covered	Mechanistic Analysis	In Vitro–In Vivo Gap	Infection-Specific Framework	Clinical Translation Guidance	References
Non-antibiotic antibacterial therapies	NP, hydrogel, coatings	Partial	X	X	Partial	[23]
NP–hydrogel hybrid systems for antimicrobial delivery	NP + Hydrogel	Partial	X	X	X	[33]
Nanomaterials for biofilm inhibition	NP only	Partial	X	X	X	[36]
Surface coatings for orthopedic implants	Surface coatings only	Partial	X	X	Partial	[27]
Nanomaterial-enabled anti-biofilm strategies	NP only	Partial	X	X	X	[37]
Formulation-dependent antibacterial performance	NP, Nanofiber, Hydrogel, Coating, Microcapsule	✓	✓	✓	✓	This review

✓ = addressed; X = not addressed; Partial = partially addressed.

**Table 2:** Comparative overview of five major antibacterial formulation platforms, summarizing key advantages, primary limitations, dominant antibacterial mechanisms, and representative clinical applications.

Formulation Platform	Key Advantages	Primary Limitations	Dominant Antibacterial Mechanisms	Representative Clinical Applications
Nanoparticle-based Systems	High surface area; tunable size and surface chemistry; enhanced bacterial membrane interaction; barrier penetration capability; stimuli-responsive drug release	Rapid clearance in vivo; protein corona formation; potential cytotoxicity; aggregation in biological fluids; limited spatial retention	ROS generation; membrane disruption; metal ion release; intracellular delivery. Mechanism driven by direct bacterial contact and high local antimicrobial concentration	Acute wound infection treatment; topical antimicrobial delivery; systemic infection management; combination therapy with antibiotics
Nanofibrous Scaffolds	High porosity; structural support for tissue regeneration; sustained local antimicrobial release; large surface-to-volume ratio; adaptable to wound geometry	Limited deep tissue penetration; mechanical fragility; potential for rapid degradation; complex manufacturing at industrial scale	Contact-mediated killing via surface-immobilized antimicrobials; sustained release of antibiotics or metal ions. Mechanism governed by interfacial contact and structural retention at wound sites	Chronic wound dressings; tissue engineering scaffolds; burn wound management; post-surgical infection prevention
Hydrogel Matrices	Sustained and controlled antimicrobial release; moisture retention beneficial for wound healing; microenvironment modulation; injectable formulation possible; supports tissue regeneration	Limited deep penetration into infection sites; potential mechanical weakness; degradation rate variability; complex crosslinking optimization	Sustained antimicrobial release; stimuli-responsive release triggered by pH, enzymatic activity, or ROS. Mechanism dominated by controlled drug diffusion and prolonged local exposure rather than direct bacterial contact	Chronic infected wound management; diabetic ulcer treatment; injectable depot for deep tissue infections; post-operative infection prevention
Surface Coatings	Localized surface protection; long-term antifouling capability; direct integration with implant surfaces; no systemic drug exposure; mechanical durability	Limited to surface-level protection; insufficient for treating established biofilm infections; coating delamination risk; limited drug reservoir capacity	Contact-mediated killing; antifouling via surface modification; localized drug elution. Mechanism relies on direct surface–bacteria interaction, making it highly effective for infection prevention but limited against established biofilms	Orthopedic and dental implant infection prevention; catheter-associated infection prevention; medical device surface protection
Microcapsule- and Vesicle-Based Systems	High drug loading capacity; protection of encapsulated agents; infection-responsive triggered release; co-encapsulation of multiple agents; membrane-mimicking structure for enhanced bacterial interaction	Complex synthesis and scale-up; stability issues during storage; potential premature drug release; limited tissue penetration in dense infection environments	Controlled and triggered antimicrobial release; barrier penetration via membrane fusion. Mechanism driven by stimuli-responsive shell degradation in response to infection-associated pH, enzymes, or ROS	Deep tissue and abscess infection treatment; biofilm-targeted delivery; combination antimicrobial therapy; localized depot injection

**Table 3:** Formulation-selection framework for antibacterial biomaterials based on infection scenario, key evaluation metrics, and design principles.

Infection Scenario	Key Evaluation Metrics	Preferred Formulation	Dominant Mechanism	Design Principles
Acute wound infections	Log reduction ≥ 3 log CFU/mL (planktonic); rapid bactericidal onset	Nanoparticle-based systems; burst-release hydrogels	Membrane disruption; ROS generation	Barrier penetration; Microenvironment matching
Chronic infected wounds	Biofilm log reduction; MIC shift under biofilm conditions; penetration depth through EPS	Hydrogel matrices; nanofibrous scaffolds; hybrid systems	Sustained release; biofilm penetration	Spatial retention; Dynamic responsiveness
Implant-associated infection prevention	Anti-adhesion rate; surface log reduction; long-term antimicrobial durability	Surface coatings; antifouling layers	Contact-mediated killing; antifouling	Architecture–mechanism coupling; Host compatibility
Established implant infections	Biofilm penetration depth; MIC shift; sustained drug release kinetics	Infection-responsive coatings; hydrogel depots	Triggered release; biofilm disruption	Barrier penetration; Spatial retention
Deep tissue infections	Drug retention at site; sustained release profile; local vs. systemic concentration ratio	Injectable hydrogels; microcapsule carriers	Sustained release; triggered release	Spatial localization; Translational feasibility

## Data Availability

No new data were created or analyzed in this study.

## Abbreviations

The following abbreviations are used in this manuscript:

AMR	Antimicrobial resistance
ROS	Reactive oxygen species
MIC	Minimum inhibitory concentration
